# Acute interstitial nephritis in the south of Iran; an observational study

**DOI:** 10.15171/jnp.2017.37

**Published:** 2017-04-20

**Authors:** Zohre Khodamoradi, Maryam Pakfetrat, Simin Torabinezhad, Mohammad Mahdi Sagheb

**Affiliations:** ^1^Student Research Committee, Shiraz University of Medical Sciences, Shiraz, Iran; ^2^Department of Nephrology, Nephro-Urology Research Center, Shiraz University of Medical Sciences, Shiraz, Iran; ^3^Department of Pathology, Shiraz University of Medical Sciences, Shiraz, Iran

**Keywords:** Acute interstitial nephritis, Steroid treatment, Kidney biopsy, Acute kidney injury

## Abstract

**Background:**

Acute interstitial nephritis (AIN) is an emerging cause of acute kidney injury (AKI) during the recent years.

**Objectives:**

There is no data about prevalence, causes, clinical manifestation and outcomes of AIN in our region. Hence, in this study we aimed to find the prevalence of AIN and describe the causes, clinical presentation, and the outcome of AIN in the native kidney biopsies.

**Patients and Methods:**

We reviewed 934 native kidney biopsies from 2006 to 2014 and collected the data of patients with the diagnosis of AIN including medical history, clinical findings, para-clinical data, pathologic findings, treatment and outcomes.

**Results:**

Prevalence of AIN in our center during 2006 to 2014 was 2.5% of all renal biopsies. The common cause of AIN in our study was drugs. Of those patients admitted to hospital due to AIN, 17 patients (70.8%) received corticosteroid, five of them (29.4%) received pulse of corticosteroid, and 12 patients (70.6%) received oral drug. Around, 54.2% of the patients had hemodialysis during admission. Eight patients had received both dialysis and corticosteroid. Two of them (8.3%) remained on dialysis and 8 (33.3%) developed chronic kidney disease, but 14 (58.3%) patients recovered.

**Conclusions:**

The prevalence of AIN in our study is comparable to other studies and we found the great impact of medications on development of AIN.

**Implication for health policy/practice/research/medical education:**

Acute kidney injury (AKI) is an increasing problem across the world. Acute interstitial nephritis (AIN) is a main cause of AKI during the recent years and also it has an increasing prevalence. AIN is a major health problem and is associated with morbidity and mortality. Our findings showed that prevalence of AIN in our center during 2006 to 2014 was close to that in western countries. Nowadays etiology of AIN has changed and drugs became an emerging cause. Antibiotics and proton pump inhibitors (PPI) are the major culprit in drug- induced AIN. Although there are controversies regarding efficacy of corticosteroids for the treatment of AIN, corticosteroids are used in AIN.

## 1. Background

Acute interstitial nephritis (AIN) is an inflammatory renal parenchymal disease defined as a specific kidney injury by Councilman in 1898 (1-3). It is now known to be a serious cause of acute kidney injury (AKI). Its prevalence has globally increased during the recent years (1-5). This is caused by immune response to some stimuli, which is characterized by interstitial edema, interstitial inflammation, and tubulitis, which causes an acute decrease in renal function(1,4,6-8). In some studies, prevalence of AIN is 1%–3% of all kidney biopsies; however, when it was limited to patients with AKI, it accounted for 15%–27% (2,4,9). With subtle clinical manifestation and empirical treatment after bedside diagnosis and without kidney biopsy, the exact incidence of AIN remains unknown. Nowadays, drugs are the major culprit in acute interstitial injury (2,4). Nonsteroidal anti-inflammatory drugs (NSAIDs) are a considerable cause of AIN surpassing antibiotics as the most important cause in some reports. Proton pump inhibitors (PPI) are gaining importance as the emerging cause of AIN in recent studies (2-4,10-12).

Autoimmune disease and recently described IgG4 related disease are other causes of AIN, and in some series no clear cause of interstitial nephritis has been revealed (2,3,13-15).

The typical signs of AIN are fever, rash, and peripheral eosinophilia. Other clinical features caused by renal failure and uncommon symptoms are found in patients with AIN (1,2,4,10,16). There is a controversy about the benefit of steroid in the treatment of AIN. Many studies have reported very good outcome in patients treated with steroids; however, some studies have not shown any effectiveness (1-5).

## 2. Objectives

Presently we have few objective data about prevalence, causes, clinical manifestation of AIN and the outcome of patients in our region. Hence, in this study we aimed to find the prevalence of AIN and describe the causes, clinical presentation, and the outcome of AIN in the native kidney biopsies over a 9-year period.

## 3. Patients and Methods

### 3.1. Study population

From all 2433 patients admitted with diagnosis of AKI in Namazee hospital and 934 native kidney biopsy reports between 2006 and 2014, pathologic diagnoses of AIN were collected. All biopsies were taken in Namazee hospital, the only educational hospital offering kidney sampling in Fars province. The pathologic reports available in Shiraz Kidney Research Centre were used in this study. We excluded kidney transplanted biopsies and patients younger than 16 years of age.

### 3.2. Clinical data

We gathered data by chart review and the records of patients were reviewed for demographic data, medical history, clinical findings, laboratory data, pathologic findings, treatment of AIN and the outcomes.

The majority of those who were diagnosed with AKI biopsy were used. AKI Network (AKIN) criteria classified the patients into three stages of AKIN. The first stage is define as serum creatinine increase ≥0.3 mg/dL or increase to 1.5–2.0-fold from baseline; the second stage is serum creatinine increase >2.0–3.0-fold from baseline and the third stage is serum creatinine increase >3.0-fold from baseline or serum creatinine ≥4.0 mg/dL with an acute increase of at least 0.5 mg/dL or need for RRT (renal replacement therapy) (17-21). Leukocytosis was defined as leukocytes >10.5 × 10^9^/L, eosinophilia as eosinophils >0.5 × 10^9^/L. Proteinuria as protein (>150 mg/d) and anemia was defined as Hb< 13 g/dL in males and < 12 g/dL in females.

### 3.3. Pathologic studies

For diagnosis of AIN, tubulointerstitial nephritis is characterized histologically by inflammation of interstitium and tubules. Tissues were stained by hematoxylin and eosin, periodic acid–Schiff (PAS), Masson’s trichrome, and Jone’s methenamine silver.

### 3.4. Outcome

The outcome is based on the interview after discharge with patients who recovered and those who did not. We defined recovery as normal serum creatinine, less than 1.5 for men and 1.4 for women and no need to use drugs or dialysis. No recovery defined as patients who became end-stage renal disease (ESRD), had kidney transplantation, needed dialysis or continued steroid therapy or with higher creatinine.

### 3.5.Ethical issues

1) The research followed the tenets of the Declaration of Helsinki; 2) informed consent was obtained; and 3) This study was approved by the Ethics Committee of Shiraz University of Medical Sciences.

### 3.6. Statistical analysis

Qualitative variables were described using frequency (%) and quantitative variables were described using mean ± SD. Fisher exact test was used to assess the relationship between qualitative variables AKIN-stage and outcome. Student’s *t* test was used to compare the mean of qualitative variables between groups of AKIN -stage and outcome. SPSS 17.0 (Chicago, IL, USA ) was used for statistical analysis.

## 4. Results

There were 934 native kidney biopsies from 2006 to 2014; among them, only 24 adults were diagnosed with AIN, thus the prevalence of AIN in our center during this period was 2.5% of all renal biopsies. Almost all of the patients were outpatient who developed AKI acutely and at the time of admission, 75% of them were in the third stage of AKIN. Table 1 shows demographic and clinical features of these patients.

**Table 1:** Description of patients’ characteristics

**Variable**	**Descriptive index**
Age (y)	43.08±19.17
Sex	
Male	12(50.0%)
Female	12(50.0%)
Inpatients	1 (4.2%)
Duration of hospitalization (days)	13.39±8.33
HTN	5( 20.8%)
Fever	9 (37.5%)
Rash	1 (4.2%)
Arthralgia	1 (4.2%)
Flank pain	7(29.2%)
Hematuria (microscopic) (>3 RBC)	17(70.8%)
Hematuria (gross)	6(25.0%‏(
Cr (admission)(mg/dL)	5.65±2.81
Cr (peak)(mg/dL)	6.24±3.59
Urine pro.24(mg/24 h)	813.16±1776.55
ESR (mm/h)	67.84±28.94
CRP(mg/L)	44.80±38.99
BUN (mg/dL)	55.66±33.02
Eosinophilia (c 0.5 × 10^9^/L)	1 (4.2%)
Anemia	20 (83.3%)
Leukocytosis (>10.5 × 10^9^/L)	5 (20.8%)
Pyuria (>5WBC/HPF)	15 (62.5%)
Proteinuria(>150 mg/d)	14 (58.3%)
AKIN stage	
Stages 1&2	6 (25.0%)
Stage3	18 (75.0%)
Infiltration	
Mild	11 (45.8%)
Moderate	3 (12.5%)
Severe	10 (41.7%)
Interstitial fibrosis	9 (37.5%)
Granuloma	1 (4.2%)
Tubular necrosis	5 (20.8%)
Interstitial edema	17( 70.8%)
Treatment	
Dialysis	13 (54.2%)
Steroid	17 (70.8%)
PO	12 (70.6%)
IV	5 (29.4%)
Recovery	
Complete	14 (58.3%)
Partial or none	10 (41.7%)
CKD	8 (33.3%)
ESRD	2 (8.3%)
Ultimate outcome	
Normal	14 (58.3%)
CKD	8 (33.3%)
ESRD	2 (8.3%)

Quantitative variables were described using means ±SD and qualitative variable were described using frequency (%).

Amongst these 24 patients included in this study, the sex distribution ratio was equal with an average age of 43.08 ± 19.17 years. These patients’ mean creatinine was 5.65±2.81 mg/dL at the time of admission due to AKI. About one third of the patients were febrile; however, rash, arthralgia and infection were rare. None of them had triad of fever, eosinophilia and rash. Just five patients had HTN at the time of admission. More than half of the patients had proteinuria and only one of them had nephrotic proteinuria. Seventeen patients had microscopic hematuria and six of them had gross hematuria and 15 patients had pyuria. The causes of AIN in this study are listed in Table 2.

**Table 2:** Causes of AIN

Causes	**No.**	**Percent**
Antibiotics	**4**	**16.7**
NSAID	**5**	**20.8**
PPI	**4**	**16.7**
Idiopathic	**4**	**16.7**
Infection	**1**	**4.2**
Bee bite	**1**	**4.2**
Anabolic androgenic steroids	**2**	**8.3**
Opium	**1**	**4.2**
Myoglobin	**1**	**4.2**
Warfarin	**1**	**4.2**

The common causes of AIN in our study were drugs; the three most common culprit drugs were NSAID (20.8%), antibiotics (16.7%) and PPI (16.7%).

Of those patients admitted to hospital due to AKI with impression of AIN, 17 (70.8%) patients received corticosteroid; five (29.4%) patients received pulse of corticosteroid, and 12 (70.6%) patients used oral corticosteroid. About 54.2% of the patients had hemodialysis during admission. Eight of them had received both dialysis and corticosteroid. No difference in the outcome was detected between the two groups of treatment.

## 5. Discussion

AKI is an increasing problem in both high and low income countries nowadays (1-3). Although etiology and presentation of AKI are different, the overall incidence of AKI is about 2%-3%. AIN accounts for 1%-2% of native kidney biopsies in some studies but in patients with AKI undergoing renal biopsy, AIN forms (constitutes) 15%-27% of the cases (2-4).

Decreasing threshold for early renal biopsy in patients with AKI tend to improve the diagnosis of AKI and increase the incidence of AIN (22).

In our region, AIN is also a major health problem and is associated with morbidity and mortality. Ossareh et al showed that the prevalence of AIN in a renal biopsy series is about 2% (9). Prevalence of AIN in our region is close to that in European and North American countries (2,15).

Prevalence of AIN in our center during this period was 2.5% of all renal biopsies. Seventy-five percent of patients were in the third stage of AKIN at the time of admission, thus we can assume most of the patients had severe AKI and majority of them (54.1%) needed dialysis treatment.

AIN at first was diagnosed through infection (3,4,10). In addition to increasing the prevalence of AIN, nowadays its etiology has changed (6,15). Even though, infection was the most common cause of AIN, presently most studies showed drug induced AIN has become the main type of AIN and antibiotic is the number one culprit (1,4,15). However, recently NSAID surpassed it as the cause of AIN. PPIs has been gaining position on the NSAID and it will surpass it in the near future (2,10,15). Elderly people are more susceptible to PPI induced AIN and this prototype of AIN is milder than antibiotics and do not have any typical symptoms (2,15,23).

In our study, drugs are the first cause of AIN (66.7%) and the three most common drugs were NSAID (20.8%), antibiotics (16.7%) and PPI (16.7%). We realized that from all four patients with AIN due to PPI, three were older than 54 years of age, but we didn’t find any differences between stage and severity of AKI in PPI induced AIN and other types. Although autoimmune disorders such as sarcoidosis, Sjögren’s syndrome and IgG4 related disorders are the important causes of AIN (2,13-15). In our cases we did not have any autoimmune related AIN.

The typical signs of AIN are fever, rash, and peripheral eosinophilia. Other clinical features caused by renal failure and uncommon symptoms are found in patients with AIN (1,4,10,15,16). Michel and Kelly showed less than 30% of patients had classic triad of AIN (10). In our study, we did not find the classic triad of AIN (fever, rash and eosinophilia) in any of the patients. Our cases had non-specific symptoms. Around, one third of them had fever at the time of admission. Pyuria was diagnosed in 62.5% of patients. More than half of them had proteinuria at time of admission; just one patient had nephrotic syndrome. Microscopic hematuria was the most common laboratory manifestation in our patients and 25% of patients had gross hematuria. Twenty percent of our patients had hypertension and all cases had some sort of AKI. Eighteen (75%) patient had severe form of the disease, of whom 13 (54%) patients needed dialysis therapy.

Goicoechea et al showed that 85% of their patients had AKI, but there is not any data about dialysis requirement in their series but in the pooled data from González et al, and Clarkson et al, 40% of patients needed dialysis treatment while all of them had AKI (1,24).

Immunologic aspects of AIN mandate to discontinue the probable culprit drugs and advocate the use of corticosteroids as therapeutic consideration although its efficacy is very controversial. Some studies questioned the effectiveness of corticosteroids. Clarkson et al showed no benefit of corticosteroid therapy. In contrast, González et al found good recovery of kidney functions after treatment with steroids (1,2,4,5,10,24-26).

In our study, 17 (70.8%) patients took corticosteroids. We could not show any difference in recovery from AKI disregarding whether they were treated with corticosteroids, mostly due to small proportion of patients and short duration of treatment.

The majority of patients – 14 (58%) – recovered from AKI completely without any residual kidney dysfunction after one year follow up. Two (8.3%) patients remained dialysis dependent and 8 (33.3%) recovered partially with some degree of chronic renal damage but free from dialysis.

As our findings, Muriithi et al showed 54% recovery in AIN patients. Four percent of their patients developed ESRD and 42% progressed to chronic kidney disease (CKD) (3).

It is important to note that despite recovery of AKI in the majority of patients, a significant proportion of patients progressed to CKD consistent with data revealed any episode of AKI puts the patient at increased risk of CKD (27,28).

## 6. Conclusions

In this study prevalence of AIN was close to that in other studies. We showed that drugs are the first cause of AIN. We also found microscopic hematuria was the most common laboratory manifestation in our patients.

## Strengths and limitations of the study

Our study is the first in Iran, showing the clinical manifestation and outcome of patients with AIN. The proportion of our cases is not sufficient to show the effect of corticosteroids and clinicopathologic findings on renal outcomes. One of the most important limitations of our study was its retrospective design which may cause an uncontrolled nature of information. Patients with mild AKI and subtle clinical manifestation may not undergo kidney biopsy, hence, they were not included in our series. However, we showed that AIN is an emerging disease entity and new etiologies such as PPIs and androgenic steroids may contribute to its increasing trend.

## Acknowledgments

We would like to thank Shiraz University of Medical Sciences, Dr. M. Vosoughi and Dr. L. Malekmakan.

## Conflicts of interest

The authors report no conflicts of interest.

## Authors’ contribution

ZKH, MP and MMS conducted the research. ZKH and MMS ana­lyzed the data. ZKH, MP, ST and MMS prepared the primary draft. ZKH, MP, ST and MMS edited the final draft. All authors signed the manuscript.

## Funding/Support

This project funded by research deputy, Shiraz University of Medical Sciences and extract from Zohre Khodamoradi M.D. thesis (Thesis # 9301017755).
